# Responsible Adoption of Artificial Intelligence (AI) in Pharmacy Practice: Perspectives of Regulators in Canada and the United States

**DOI:** 10.3390/pharmacy13060152

**Published:** 2025-10-27

**Authors:** Paul A. M. Gregory, Zubin Austin

**Affiliations:** Leslie Dan Faculty of Pharmacy, University of Toronto, Toronto, ON M5S 3M2, Canada; paul.gregory@utoronto.ca

**Keywords:** artificial intelligence, pharmacy practice, health professionals’ regulation, pharmacy regulators

## Abstract

Background: Use of Artificial Intelligence (AI) is proliferating in society and in pharmacy practice. For some, this represents a great advancement that will enhance effectiveness and efficiency of health care. For others, it is an existential risk that will worsen inequalities, lead to deskilling of the workforce, and spiral beyond the comprehension or control of humans. Human-in-the-loop (HiL) vs. human-out-of-the loop (HoL) AI have different potential risks and challenges that raise questions regarding patient safety. Defining principles for responsible adoption of AI in pharmacy practice will be an important safeguard for both patients and the profession. Methods: Semi-structured interviews with 12 pharmacy regulators from across Canada and the United States were undertaken, with informed consent. Constant comparative data analysis using nVivo v15 was used to identify common themes. The COREQ framework was applied to assure quality of research processes used. Results: Pharmacy regulators highlighted the value of a principles-based, rather than rules-based, approach to AI. Core principles related to transparency, redundancy, audit and feedback, quality assurance, privacy/data security, alignment with codes of ethics, and interoperability were identified. There was limited consensus on the role of consent and choice as principles to be considered. Conclusions: The role of regulation in shaping responsible adoption of AI in pharmacy will be significant. This study highlighted a series of agreed-upon principles but also identified lack of consensus with respect to how consent and choice could be operationalized in pharmacy practice.

## 1. Introduction

Microsoft Copilot (a widely available AI-powered assistant integrated into commercially available, ubiquitous software) describes Artificial Intelligence (AI) as “…a branch of computer science focused on creating systems that can perform tasks typically requiring human intelligence” [1]. It further enumerates five specific facets of human intelligence: learning, reasoning, perception, language understanding, and autonomous action.

Since the launch of ChatGPT in late 2022, the power of AI has captured both public imagination and generated a dizzying array of questions, concerns, and opportunities [2,3]. Within a few short years, AI has become embedded in daily life in ways that many ordinary people may not be aware of [4]. Smartphones, appliances, automobiles and many other consumer purchases now utilize many different forms of AI [5]. More recently, governments around the world have touted use of AI as a tool for reducing costs, enhancing efficiency and improving effectiveness of all manner of public services [6,7]. Within education, the rapid proliferation of AI is fundamentally shifting the nature of teaching, learning, and testing in ways that may be shifting our understanding of concepts such as “critical thinking” or “persuasive writing” [8,9].

In the work world, AI has already re-shaped the roles and responsibilities of diverse professions including engineering and accounting [10]. Within health professions, the spread of AI is also gaining momentum [11].

The scope and speed of AI’s evolution have some uncertainty and confusion around its definition. Microsoft Copilot defines three different types of AI [1]: (a) Narrow AI, designed for a specific task (e.g., responding to voice commands from humans); (b) General AI, or systems that can perform intellectual/cognitive tasks at a level equal or greater to humans (e.g., decision support for prescribing medications); and (c) Generative AI, capable of actually synthesizing new knowledge or new content in diverse media ranging from text to images to music (e.g., Chat GPT). A more human-centered typology for describing AI categorizes it based on the extent to which ultimate decision-making control rests with the human or rests with the AI [12]. Human-in-the-loop (HiL) AI involves systems that may advise, suggest, or recommend but ultimately a human must make the final decision and actively perform an action (including entering a key stroke on a computer) to enact an outcome. Within the pharmacy world, HiL systems may include drug-interaction detection or prescribing decision support where the AI may review, analyze and recommend, but still requires a human agent—the pharmacist—to make the final decision [13]. In contrast, there are an increasing number of human-out-of-the-loop (HoL) AI systems that are proliferating, in which final decision making and enaction no longer require a human being, and indeed, human actions and wishes can in some cases be explicitly ignored or overruled by the AI itself. For example, in some autonomous vehicles with certain kinds of collision detection or braking systems, the AI makes the decision and even if the human disagrees, there is no way to override the system’s power [14].

Within health professions, the distinction between HiL and HoL AI has been a crucial marker of human oversight and control, reassuring both patients and professionals of the continuing importance and value of human judgement and skills [15]. As AI has proliferated, however, this distinction has become less clear and significant issues have emerged. The term “deskilling” describes the process by which trained human professionals (such as pharmacists) become ever-more reliant on HiL AI over time, and even if a keystroke or some other kind of human intervention is required to “approve” the AI’s suggestion, rote automaticity of human action simply, mindlessly, and without intentionality approves whatever the AI suggests [16,17]. While HiL AI gives the illusion of human oversight, as a workforce becomes progressively deskilled, this oversight is merely theatrical, not actual. In this way, AI that is HiL in name and by design, ends up being operationalized as HoL in reality [18]. Consider for example, the ways in which the proliferation of a much simpler technology—calculators used for math—have eroded society’s capacity to undertake simple mathematical calculations (or “mental math”). At one time, most adults could go to a grocery store and tally up the cost of their purchases in their head to know if they had enough money to buy all they wanted. Today, few teenagers or young adults have the capacity to engage in simple mental addition such as this—a sobering example of the power of deskilling brought about by a much simpler technology (calculators) than AI.

Many other concerns have been raised regarding the rapid proliferation of AI in healthcare and beyond. Dystopic science fiction has long feared the spread of “thinking machines” that would eclipse human intelligence and enslave humanity—HAL the shipboard computer from the movie 2001: A Space Odyssey or Skynet from the Terminator are key examples. The notion that AI could replace an increasingly deskilled workforce also raises issues in terms of professions and their structure, particularly in the context of health care where the need for cost-effectiveness and cost-constraint may lead some administrators to wonder whether human workers are worth their costs [19]. Concerns have also been expressed about the ways in which AI may further widen societal disparities and accelerate political polarization [20,21]: for example, already in education, a two-tier system is evolving where students who are wealthy enough to afford paid subscriptions to services like ChatGPT have access to an abundance of resources that are not available to those who can only afford the unpaid free version of that system. Finally, the science of AI is itself an enormous, multi-national for-profit business. Some of the most powerful and wealthiest companies and people in the world are driving the proliferation of AI, and they are doing so explicitly for the purpose of making money. With respect to the public interest and social cohesion—how trustworthy is this architecture? In the past 20 years, we have had experience with a far less sophisticated technology—social media (such as Facebook)—and how it has fundamentally changed society, politics, government, and daily life. No power, not even the United States government, is able to contain or control the implications of that technology despite documented harms that have resulted. The nature of AI is far more complex, far more sophisticated, and far more impactful—yet attempts to regulate and control malign outcomes have been limited, in the name of not stifling free speech or innovation [22].

Traditionally, when new or potentially damaging goods or services are introduced into the marketplace, regulation has been seen as an important safeguard for public protection. In the pharmacy world, agencies such as the European Medicines Agency or the Food and Drug Administration use expert-driven, objective, transparent, and robust assessment systems to evaluate safety and efficacy from the perspective of the general public and monitor compliance. Manufacturers are required to adhere to standards and respond to regulatory direction. Within the context of AI—which, ultimately is a manufactured good/service within a for-profit context—regulation has been challenging to enact. The European Union has attempted to introduce frameworks and guiding principles, but these continue to be challenged politically; further, the accelerated pace of evolution of AI itself means that regulations may be outdated even before they are promulgated [23]. Philosophically, the United States has been opposed to regulation seen as unnecessary to potentially stifling to innovation and profit-making [24]. Indeed, in this current time, the current US government is openly disdainful and hostile towards regulation and committed to deregulation across vast swathes of society in the name of unleashing innovation. Importantly, social media such as Facebook evolved in a similar regulatory vacuum and was initially viewed as a curiosity, or a toy—but now its implications are much better understood. The current evolution of AI within a similar regulatory vacuum raises concerns and questions regarding what will happen in the years ahead.

In Canada and the United States, “regulation” of health professions is an essential safeguard for public interests. The complex and challenging work that professionals like pharmacists do requires close oversight to ensure it is done safely, effectively, efficiently, and in alignment with ethical standards. Licensing or regulatory bodies use a variety of legalistic tools (regulation) to ensure this objective is achieved through direct oversight and monitoring of individual professionals (e.g., through competency assessment requirements at entry to practice and throughout the career of a practitioner). While there are different philosophies between jurisdictions regarding how regulatory bodies can achieve this objective, and different degrees of power/authority conferred by government with respect to self-regulatory independence of professionals, Canada and the US broadly share similar approaches and tools.

Beyond these fears, however, there are significant opportunities that new technologies such as AI can bring. While the promise of AI may be overhyped at times, it is still significant. The power of AI in health care is only now being explored, in areas ranging from personalized counselling for smoking cessation and mental health services to personalized medicine/pharmacogenomic treatments to more cost-effective inventory management. In financially constrained health systems, AI may be the only and best option to continue to deliver high quality care. The accuracy and quality of AI usually exceed that of any individual human [25], and concerns that its role in health care is limited because patients always want a human touch may be changing rapidly [25,26].

In this context, pharmacy professionals in both community and hospital settings are using and introducing AI-powered and AI-supported tools in their practices in myriad ways—most of which are unregulated and unmonitored. Concerns regarding deskilling, exacerbation of societal inequalities, and being captive to single technologies exist, but do not appear to be slowing down adoption of AI in practice. The licensing and regulatory bodies that oversee the practice of pharmacy have statutory responsibilities to safeguard public interests and ensure safe and effective practice of the profession—but what does this actually mean and look like in an era of HiL and HoL AI? The public and the profession rely upon regulatory bodies to establish the ground rules for safe and effective practice and to define the perimeter of responsible professional work—yet few if any pharmacy regulators have developed polices or regulations to guide the profession in responsible adoption of AI in practice.

The objective of this research was to explore regulators’ perspectives on responsible adoption of AI in pharmacy perspective, as a way of opening reflection, discussion, and ultimately action to support the pharmacy profession in continuing to deliver safe and effective care in this era of rapid proliferation of both HiL and HoL AI.

## 2. Materials and Methods

There is currently scant literature describing pharmacy regulators’ perspectives on the regulation of artificial intelligence in pharmacy practice, as the phenomenon itself is relatively new and rapidly evolving. As a result, an exploratory research method was selected for this study in order to provide an opportunity to examine the issue holistically and from a stakeholder-focused perspective. Exploratory research such as this does not purport to be generalizable but instead aims to identify important themes that may warrant further in-depth examination in future studies [27]. This research was also framed around the notion of perspectives, an inherently subjective construct that involves both personal and professional experiences as well individual characteristics of participants. As a result, a qualitative research method was selected, utilizing the tool of the semi-structured interview (SSI). SSIs permit researchers to provide space for participants to discuss issues from their own subjective perspectives within the broader context of an overall research objective [28]. SSIs focus on both conversation and relationship building between participant and researcher in order to elicit thoughtful, honest, and relevant insights to achieve research objectives [28]. For this study, an SSI protocol was developed and received institutional research ethics board approval (see Appendix A), focused on the topic of responsible regulation of AI in pharmacy practice.

Participants in this research were recruited through purposive and snowball sampling methods. For this study, all participants were employed in pharmacy regulatory/licensing bodies in Canada or the United States and thus had experience with regulation of the profession. These participants did not necessarily have to be pharmacists or pharmacy technicians themselves or have any personal experience using AI in practice. Participants were recruited through several means: initial key informant outreach (purposive sampling) was undertaken based on the research teams’ professional networks. Those interested in participating in the study were then asked to nominate other pharmacy regulators in their own networks who they thought might be interested in participating (snowball sampling). This recruitment approach meant that the participants involved in this study were not necessarily representative of the pharmacy regulatory community.

Potential participants were provided with written information by email outlining the objectives and the processes involved in the study. Informed consent was sought and received prior to undertaking enrolment in the research. They were asked to participate in a 45-min Zoom-based interview at the time of their choosing. During this time, a researcher would interview them regarding their perspectives on responsible adoption of AI in pharmacy practice; with the participant’s permission, interviews would be recorded and verbatim transcripts produced for subsequent analysis. Participants would have access to their own transcripts for review if desired.

Data from transcripts would be managed for analysis using NVivo v15. All transcripts would be reviewed by two researchers who would categorize and code for emergent themes based on study objectives. Each researcher would independently review transcripts and both reviewers would meet to achieve consensus on themes that emerge. An iterative constant-comparative analytical method would be utilized to establish a coding tree that would be refined through subsequent interviews and analysis, until thematic saturation had been achieved [29]. These themes would then form the basis for reporting of research findings. Given the small number of pharmacy regulators in Canada and the United States, protecting their identity and ensuring their confidentiality during this process was a priority; all transcripts were labeled using non-descriptive alphanumeric codes rather than names, and transcripts were redacted if identifying information (e.g., a state or province name) was mentioned. Transcript and analytical data were securely electronically stored behind two-factor authentication databases. Thematic saturation—the point at which analysis of new data produced no new codes, themes or insights—was used to define the endpoint of data collection [27]. To ensure thematic saturation was accurately assessed, two additional interviews were conducted following the point where researchers believed saturation had been achieved [27].

To ensure trustworthiness and indicativeness of this research, the Consolidated Criteria for Reporting Quality Research (COREQ) system was used [30]. This 32-item checklist guides qualitative researchers throughout the process to ensure all aspects of data collection, analysis, and reporting conform to quality expectations. This research was approved by the University of Toronto’s Research Ethics Board (Protocol 46033 Approved 1 February 2024).

## 3. Results

A total of 12 pharmacy regulators from across Canada and the United States agreed to participate in interviews. They represented diverse geographical regions, including more urbanized (*n* = 8) and more rural areas (*n* = 4). Approximately one-sixth of these regulators (2/12) were themselves pharmacy professionals (i.e., licensed as either a pharmacist or a registered pharmacy technician) with personal experience of using AI in practice, in addition to their role as a regulator. Half of the participants (*n* = 6) identified as female. All participants completed informed consent prior to their interview, and all agreed to have their interviews recorded and verbatim transcripts generated.

Based on these interviews several key themes emerged (see Table 1 for indicative transcript excerpts):

**Table 1:** Key Theme Data Table.

Theme	Indicative Transcript Excerpt
The use of human-out-of-the-loop AI in pharmacy practice is not a focus of regulatory interest; the use of human-in-loop AI is of interest to regulators.	*“Well, we can’t be in the business of regulating machines, only regulating people. So, no, if it’s human out of the loop AI you’re talking about, I can’t see how [regulatory bodies] could ever do that.”* *“We regulate professionals, so if there is a professional, like a pharmacist, involved and whether it was with the AI or not, then yes, the regulator has a legitimate interest in protecting the public and investigating if a patient is harmed. But not if it is just an AI related issue on its own.”*
The use of “regulation” to direct responsible adoption of AI in pharmacy practice may not be feasible; instead, the use of “guidance” was preferred.	*“People often think regulation is the answer to every problem, but believe me, it really is not. Regulation is a pretty blunt instrument so only needs to be used when there’s really no other option. We don’t And I think with AI—there’s lot of other options that need to be considered first.”* *“Honestly, I don’t actually see how regulation could even work with respect to AI. These companies—I mean you’re talking like Google and Microsoft—huge and powerful international businesses. We’re just a regulatory body, how can we possibly deal with them? Besides any time regulation is mentioned with AI, we worry we will be seen as the villain, slowing things down or inappropriately interfering.”*
Key principles of guidance focused on AI in practice included: transparency, redundancy, audit and feedback, quality assurance, privacy/data security, alignment with codes of ethics, and interoperability.	*“What we need—what regulators need—is some kind of list of different principles that we can use to guide pharmacists in making good choices. Things like privacy or data security, you know things they need to be aware of before selecting and using AI.”* *“The most important thing we can do is to provide a checklist so pharmacists know how to make the best choices for themselves. Things like reminding them about the code of ethics, or ensuring they are being open with patients when they are using AI—these kinds of checklists would be so helpful, for us as regulators and for the profession.”* See Table 2: Key Principles Data Table for additional transcript excerpts
There was no consensus on the issue of “informed consent” or “choice” for patients with respect to use of AI in pharmacy practice due to operational/logistical issues.	*“Choice? Consent? Yeah these must be the thorniest issues to deal with. Of course—well, regulators have always supported, actually required, informed consent and choice for patients. But with AI—I struggle to see how that could work? I mean just practically, could a patient opt-out of AI reviewing for drug interactions for example? It just doesn’t seem feasible.”*

Italicized text represents verbatim transcript excerpt from participant interview.

**Table 2:** Key Regulatory Principles Data Table.

Principle	Indicative Transcript Excerpt
Transparency	*“A critical principle—pharmacists need to be open, transparent, and let patients know when they are using AI for decisions, how they are using AI, and how AI might affect what care they provide.”* *“Being open and being clear with patients that AI is being used in decision making or actions that affect them—that’s essential.”*
Redundancy	*“From a safety perspective, having back-up systems is critical. Things can’t just stop if the AI isn’t working, so ensuring that the staff—and this speaks to that problem of de-skilling again—we need to make sure pharmacies can still function if something breaks down or isn’t available.”* *“No question, this is a problem of our time. How can we make sure safe and effective patient care can occur even if there is a technology breakdown? Yes, that’s the kind of principle, regulatory principle, we need to ensure.”*
Audit and Feedback	*“We ensure continuing competency of our pharmacy professionals—we need a similar system for audit and feedback to monitor AI too.”* *“Monitoring, measuring, feedback—this system is common in health professions, and should be applied to AI as well.”*
Quality Assurance	*“Regulators spend a lot of time and energy thinking about quality assurance, quality improvement. AI needs to be subject to similar scrutiny on this as human professionals.”* *“There’s so much talk about AI hallucinations, algorithm bias, that sort of thing. Quality assurance especially with decision support, decision making—that’s a critical principle for regulators to embrace.”*
Privacy/Data Security and Integrity	*“Well I’d hope that the [national] privacy, data security, that kind of legislation—that should be the floor, the bare minimum with respect to AI—as a regulatory principle, we need to aim higher than the bare minimum of the law.”* *“Who is using the data? Who is seeing the data? Who is profiting from the data? We need regulatory guidance and principles on this—I guess we need to be the ones to develop these principles actually.”*
Alignment with Code of Ethics	*“We have a code of ethics for our profession—this should apply to AI as well.”* *“The Code of Ethics—that’s the place to start in terms of principles. Everything needs to be consistent with the Code of Ethics to ensure safety for patients, the public.”*
Interoperability	*“We are going to need to be really careful to ensure we don’t end up with technology company monopolies—if that happens, professional autonomy, independence will be out the window and that’s a public protection problem.”* *“AI systems need to be interoperable, speak and connect with one another, otherwise there’s too much risk of data erosion and error. This should be a key regulatory principle I think.”*

Italicized text represents verbatim transcript excerpt from participant interview.

The use of human-out-of-the-loop AI in pharmacy practice is not a focus of regulatory interest; the use of human-in-loop AI is of interest to regulators.

All participants in this research were familiar with different forms of AI, and all reported having used AI to some degree in their own work as regulators, and/or in their work as a pharmacist or regulated pharmacy technician. They recognized and understood the distinctions between HiL and HoL AI. Amongst all participants there was a consistent perspective that pharmacy regulators only had authority over people, and not machines; consequently, where HoL AI was being used, it was not appropriate or feasible to consider regulatory means to address potential safety issues. The structure of regulation focuses strongly on named individual, identifiable practitioners over whom regulators have certain powers. Where these individuals used HiL AI—and therefore still had ultimate decision-making control—there was a legitimate basis for regulatory interest in how the HiL AI was being used. Where workplaces or organizations used HoL AI to perform activities that may have normally been performed by pharmacists or regulated pharmacy technicians, but the AI had ultimate decision-making control, this was not seen by participants as being a legitimate basis for regulatory involvement. Instead, they suggested, other routes would need to be explored in order to achieve patient protection objectives (e.g., sue the manufacturer of HoL AI in court, or government legislation rather than professional regulation). When asked about the risk of deskilling, or situations where HiL AI functions, in reality, as HoL AI, participants responded that wherever a human professional (pharmacist or regulated technician) had the opportunity to verify or override AI’s decision and did not do so, there would be a legitimate pathway for regulatory involvement in the situation. Where the technology did not permit human override (as in HoL AI), there was no basis for regulatory involvement regardless of the consequences for patients.

Participants in this study were unanimous and strong in their perspective that only humans—and not technologies—were the legitimate interest of regulatory bodies. While this perspective is understandable and defensible, it highlights an important regulatory gap that participants themselves recognized, particularly in the context of professional deskilling brough about by reliance upon AI. When probed further to describe how this gap could be reconciled given regulatory bodies stated mandates to “serve and protect the public’s interests”, participants acknowledged the issue but described this as the responsibility of governments, courts, or manufacturers’ standards associations rather than health professional regulators.

1. The use of “regulation” to direct responsible adoption of AI in pharmacy practice may not be feasible; instead, the use of “guidance” was preferred.

Participants in this study were unanimous and strong in their perspective that only humans—and not technologies—were the legitimate interest of regulatory bodies. While this perspective is understandable and defensible, it highlights an important regulatory gap that participants themselves recognized, particularly in the context of professional deskilling brough about by reliance upon AI. When probed further to describe how this gap could be reconciled given regulatory bodies stated mandates to “serve and protect the public’s interests”, participants acknowledged the issue but described this as the responsibility of governments, courts, or manufacturers’ standards associations rather than health professional regulators. While participants recognized the unique role—and power—that regulatory bodies possess in shaping and directing professional practice, they also noted the limitations to such power in the context of new technologies such as AI. A primary concern related to the power asymmetry between professional regulatory bodies and the enormous international companies currently developing AI: there was firm belief that no regulatory body, or even combination of regulatory bodies that could content with the clout of “big tech”. A secondary concern related to the perception by the profession and the public that regulation would slow down or stifle technological innovation, which would produce harm of its own in terms of compromising effectiveness or efficiency of pharmacy service delivery.

Several participants raised a broader, more existential concern with respect to the public perception of regulation. At the time of this study, the Department of Government Efficiency (DOGE) in the United States was in full force, charged with reducing administrative burden to unleash economic competitiveness [31]. Participants noted a cultural shift both within the pharmacy profession and with the general public towards a more cynical or hostile view of the societal value of regulation of any kind. The political, cultural, and social forces that had coalesced to enable the DOGE mandate created a heightened sense of vigilance with respect to the issue of regulatory over-reach. These participants noted their concern that attempting to regulate AI may appear to many as exactly the kind of regulatory over-reach that spawned the kind of cultural blowback culminating in DOGE and other similar responses. Participants noted the importance of regulators “staying in their lane” and using the blunt force of regulation only when absolutely necessary and where no other options existed.

All participants in this study believed that regulators could serve their profession and the public best by instead opting to issue “guidance”: non-binding, evidence-based suggestions to practitioners on principles for responsible adoption of AI in practice. The guidance approach stands in contrast to regulation in being informational and leaving ultimate decision-making control to individual professionals, rather than enforcement through regulation. Participants noted that guidance may appear weak or ineffectual precisely because it is non-binding, but they noted that, at this time, it was likely the only viable and acceptable option.

2. Key principles of guidance focused on AI in practice included: transparency, redundancy, audit and feedback, quality assurance, alignment with codes of ethics, and interoperability.

All participants in this study favoured a guidance-based approach, rather than a more legalistic approach grounded in regulation, to support responsible adoption of AI in professional practice. They also agreed that the rapid evolution of AI technology meant that a principles-based approach to guidance may be more appropriate rather than one that focused on micro-managing through a series of detailed rules that would be non-binding anyway. For participants in this study, the objective was to educate and empower pharmacy professionals themselves to make informed choices and decisions with respect to how and when to adopt AI in their practice, mindful of potential implications, benefits, and risks.

Participants agreed upon a series of key principles that could form the foundation of guidance documents and supports provided by a pharmacy regulator to help individual pharmacy professionals make decisions regarding responsible adoption of AI in their individual practice (see Table 3). These principles included the following:

(i) Transparency: Participants noted that it was important for both pharmacy professionals and the clients they served to be aware and make clear when and how AI was being used—was it HiL or HoL, was it being used to support decision making, or actually make independent decisions? Processes to make transparent how AI was being integrated into the practice were essential, both in safeguarding patients’ rights but in also ensuring pharmacy professionals themselves were aware of the places and ways in which AI was impacting their practice.
(ii) Redundancy: All participants described the relatively routine problem of software malfunctions, network failures, internet crashes, and other technical problems that, on occasion, leave workplaces and societies paralyzed. Even mundane everyday events such as electrical supply cuts during power failures point to the reliance workplaces and professionals have upon technologies which may from time to time fail. In this context, an important regulatory principle for AI involves redundancy of systems, particularly where HoL AI is being used, or even where HiL AI has resulted in some level of deskilling of the workforce. Backup systems that allow for service-as-normal during technology failure are an essential safeguard; for example, in the past, human operated typewriters could be used where computerized prescription systems in pharmacies were inoperable in order to allow some dispensing functions to continue. In the context of AI, “redundancy” may be more complex than that; responsible professionals must anticipate AI technology failure and ensure reasonable back up provisions exist.
(iii) Audit and Feedback: Human professionals are subject to periodic and sometimes random checks on their performance to ensure they meet quality standards and expectations with respect to both processes used and outcomes. Such Audit and Feedback systems are integral to continuous professional development and quality improvement, and similar processes were identified by research participants as an important principle for responsible adoption of AI in practice. Implicit in Audit and Feedback is the notion of monitoring and reporting in a meaningful and transparent manner so that all stakeholders are aware of the performance of AI in practice, as well as areas of concern, areas for improvement and ultimately “red flag” problem areas that require intensive attention. To allow for such Audit and Feedback, AI vendors must enable independent verification and the ability for professionals to self-generate performance reports based on measurement criteria to establish the quality and success of AI’s work in delivering pharmacy services and care.
(iv) Quality Assurance: Quality Assurance (QA) (and its related concept Quality Improvement) refers to a structured and systematic approach to ensuring ongoing enhancements of services and activities based on measurable criteria focused on specified outcomes. All human professionals have ethical requirements focused on QA and where AI is involved in work influencing patient outcomes, similar QA systems and processes need to be developed and implemented. QA typically involves measurement and comparison with benchmarking standards or expectations, along with plans to address deficiencies where they are identified. Reporting of QA activities to regulatory bodies by human professionals is commonplace; development of similar structures where AI is being deployed was identified by study participants as an important principle for responsible adoption of AI in practice.
(v) Privacy/Data Security and Integrity: In most jurisdictions, there is some kind of legislation that outlines requirements for protection of patient data and privacy. The nature of the large language models that undergird AI means that in many cases, the kind of data that is required by law to be protected for privacy is also the kind of data that is used to train AI to improve. This raises potential concerns regarding access to sensitive health data, ownership of patient records, and in the context of the for-profit companies developing AI, who financially benefits from the data AI is compiling in the course of doing its work. Further, the security of this data is essential to consider: the risk of unauthorized access, malicious hacking, or other misuse of protected data must be assured. Finally, ensuring the ongoing integrity of data, including is accuracy and preventing corruption of data files, are also important principles for responsible adoption of technologies in general, but of AI in particular.
(vi) Alignment with codes of ethics: Most professions—including the pharmacy professions—use standards of practice and codes of ethics as a tool to define minimum practice expectations associated with safe, effective and competent provision of care and services. Codes of Ethics govern a variety of topics but most often describe the reasons that underpin professional behaviours. In the context of AI, ethical concepts such as beneficence (working in the patient’s best interests), non-maleficence (first, cause no harm), justice (ensuring equitable access to health care) and respect for autonomy of the patient are all codified elements of professional practice that need to integrate in AI-driven systems. From a regulatory perspective, the same ethical expectations that govern professional-patient relationships should be embedded in AI-patient relationships and work.
(vii) Interoperability: Many participants highlighted the danger associated with reliance upon a single technology vendor and the risks of a monopoly over AI tools. Such a monopoly could pose grave risks to professional status and autonomy and directly shape a profession’s future. As a result, many participants highlighted an important principle for responsible adoption of AI that emphasized the value and importance multiple vendors to reduce risk of undue reliance on a single technological platform, particularly in the context of concerns regarding AI and deskilling of the workforce. Interoperable AI provides professionals with greater flexibility and more options and reduces risk of monopolistic practices unduly influencing professional standards of practice.

These seven guidance principles were most frequently described by participants as crucial to the articulation of a framework to guide responsible adoption of AI by pharmacy professionals. While there may be other important principles (see below) these seven were described with greatest clarity and frequency by study participants.

3. There was no consensus on the issue of “informed consent” or “choice” for patients with respect to use of AI in pharmacy practice due to operational/logistical issues

One particular issue surfaced repeatedly during the research: the concept of “informed consent” and choice with respect to whether HiL or HoL AI was being used by pharmacists. Traditionally, regulators have strongly emphasized the central roles of informed consent and choice in safeguarding patient autonomy and respecting their rights. Many participants reinforced the importance of these principles in professional practice but struggled to identify how these could be operationalized in the context of AI in practice. Participants differentiated between the “transparency” principle noted above, and affirmed its importance: communicating clearly with the patient regarding how and why AI-driven tools are being used in their care was deemed logistically feasible, but asking for permission to use them, gaining informed consent for their use, and providing an opt-out option or choice struck most participants as implausible and unworkable given pragmatic workflow considerations. This paradox caused some discomfort for many of the participants in the study, who were aware of the problematic nature of not foregrounding consent and choice as regulatory principles. Most participants agreed that this was a situation where further reflection and work was required, to identify proportionate and reasonable strategies to allow for consent and choice within the confines of the traditional workflow in pharmacy practice. Some participants expressed discomfort over the notion that pragmatic or logistical considerations were given primacy over ethical and professional priorities with respect to consent and choice but also acknowledge the pragmatic unworkability of regulatory principles that would simply be infeasible to actually implement.

In some ways, this particular theme raised a critical question at the heart of this study: the limitations of regulatory authority to address issues of ethical importance where the ethically preferred alternative introduces significant logistical challenges. AI raises all manner of issues like this that are crystallized in the debate surrounding the role of consent and choice as regulatory principles for responsible adoption by professionals. For participants in this study, this was a paradox, a problem, and a problem with no viable solution. Instead, a “least worst alternatives” approach was used to identify the most acceptable option—in this case, not including consent or choice as regulatory principles per se, but instead encouraging practitioners to—in some unspecified manner—be mindful and cautious.

## 4. Discussion

The four themes described above highlight the significant challenges faced by regulators as AI continues to evolve rapidly and integrate more deeply into daily practice of professionals and day-to-day life of the general public. On the one hand, the findings from this study may appear initially disappointing to those who believe regulators ought to exercise a more direct and interventional role in regulating AI in the public interest and to safeguard patients’ wellbeing. Participants in this study unanimously agreed that regulation was neither viable nor appropriate for a variety of reasons and instead preferred a more educational guidance-focused approach aimed at empowering professionals themselves to make informed decisions about how to responsibly adopt AI in their own practices. This approach, of course, risks fragmenting the way in which AI is adopted by pharmacists and leaves considerable scope for personal judgment that may be problematic from a patient protection perspective. It also transfers significant responsibility and blameworthiness on individual professionals and potentially leaves a regulatory vacuum that could be problematic.

This guidelines-based approach is not necessarily new with respect to socially transformative technologies. Recalling the way in which social media evolving in a similar regulatory vacuum is sobering; today, it is clear that the social media genie cannot be contained and as a result a technology promulgated by a for-profit corporation aimed at maximizing shareholder value (not at producing social progress or good) has caused some significant harm. The stance of participants in this study suggests a similarly laissez-faire attitude towards AI may also be evolving, but the societal risks posed by unregulated AI may be considerably greater than they were with social media at a similar point in its evolution. Still, as noted by the participants in this study, the plausibility of health professions regulators actually steering the direction of AI evolution seems low at best, so their stance on guidelines as opposed to regulation may be the pragmatic, least-worst alternative at this time.

Participants in this study demonstrated striking consistency in identifying seven core principles that could provide a framework to guide responsible adoption of AI by pharmacy professionals. These principles connect to other regulatory expectations—for example, codes of ethics or quality assurance requirements—and thus align effectively with the intentions and objectives of regulated health professional work. The principles described by participants balance both professional and technological risks of AI. For example, in describing the importance of redundancy to prevent service/care breakdown in case of technological failure, or in highlighting the value of interoperability to prevent monopoly control over a critical AI product, regulators in this study recognize the importance of technology itself in addition to its adoption in practice. A strong focus on privacy, data security and data integrity was a consistent theme across all study participants and highlighted potential concerns that have been previously seen in the evolution of social media but are amplified in the context of AI that is driven through large language modelling techniques. The seven principles that surfaced most clearly and frequently in this study form a potentially useful foundation for the development of educational tools and guidance documents to support pharmacy professionals in responsible adoption of AI.

The lack of clarity or consensus regarding the role of informed consent and patient choice with respect to AI highlights a central dilemma facing both regulators and practitioners. Balancing ethical principles and pragmatic operational realities, participants in this study determined that infeasible regulatory guidance would be more damaging than helpful and opted to not include these important principles in their work—for now. Acknowledging the paradoxical nature of regulators not foregrounding consent and choice as principles for responsible adoption, participants in this study highlighted these ideas as requiring further research and consensus building in the near future.

This study is amongst the first to focus on regulators as research participants, an important distinction considering the potentially large role that regulators could have in establishing the future growth and trajectory of AI in professional practice. There are limitations that must be considered that limit generalizability of findings. First, the study population—Canadian and American regulators of pharmacy practice—are not representative of the pharmacy community or the community of health professions regulators globally; the extent to which these findings can be extrapolated to another jurisdiction is not clear. Second, the purposive and snowball sampling methods used in this study—while appropriate for exploratory qualitative research examining a new and emerging issue such as this—means that even within the small frame of Canadian and American regulators of pharmacy practice, the participant pool was not representative. While the quality of data collection, analysis and interpretation was guided by the COREQ checklist approach and included appropriate safeguards including independent double coding of themes by independent reviewers and the opportunity for member-checking by research participants themselves, the narrative, qualitative approach to data gathering in this qualitative study is subject to its own subjectivities and potential biases from both participants and researchers alike. The objective of this study was not to establish globally generalizable rules for responsible adoption of AI in pharmacy but instead to open discussion, reflection, and preliminary deliberation on approaches to how best manage a massively transformational new technology already expanding rapidly in the profession and society.

Further work in this area is required as AI technology itself rapidly evolves and proliferates. In particular, further examination of the participants’ contention that “regulation” was not feasible and “guidance” was an appropriate alternative requires further examination. The power of regulation—and thus of regulators—relates to the legal strength of the concept: with regulation, sanctions, enforcement, and accountability becomes possible. The voluntary nature of “guidance” makes use of sanctions, enforcement, and accountability less impactful. This distinction suggests that the guidance approach preferred by regulators may be less effective in terms of patient protection/safety outcomes, and raises questions for regulators regarding their responsibilities. Stakeholder engagements such as this with key groups of interested individuals provide a unique and powerful opportunity to understand complex issues from multiple perspectives. In the future, research examining perspectives of AI entrepreneurs and developers, or pharmacy employers/corporations would complement work such as this and help to build a clearer understanding of the challenges and opportunities ahead with respect to integration of AI into pharmacy practice.

## 5. Conclusions

As AI technologies continue to evolve, their impact on everyday life—and the daily practice of professionals like pharmacists—will continue to grow. Speculation on the “pros and cons” of AI may be interesting but ultimately will not change the trajectory of AI’s development. As the experience of social media, a generation ago highlighted, there are potential problems when new technologies are permitted to proliferate in a regulatory vacuum. This study has explored perspectives of regulators of health professions with respect to the evolution of AI in pharmacy practice. Further work is required to continue to advance discussion, thinking, and action, to ensure AI is adopted responsibly, ethically, and in a manner that is appropriate for both the profession and those the profession serves.

## Figures and Tables

**Table 3:** Regulatory Principles for Responsible Adoption of AI in Pharmacy.

Principle	Guidance Question
Transparency	Are both patients and pharmacists aware of when and how AI is being used in care?
Redundancy	Are there back-up systems in place to manage technology failures and allow continuation of pharmacy care?
Audit and feedback	Are there monitoring systems that provide assurance of positive health outcomes associated with AI?
Quality Assurance	Are there systems comparable to those used for human professionals to measure and report quality and facilitate improvement
Privacy/Data Security/Integrity	Are safeguards that meet or exceed legislative requirements to safeguard patients interests
Alignment with Codes of Ethics	Do ethical principles that guide AI development align with those that govern human professionals?
Interoperability	Are systems in place to mitigate risks associated with technology monopolies?

## Data Availability

The original contributions presented in this study are included in the article. Further inquiries can be directed to the corresponding author.

## Abbreviations

The following abbreviations are used in this manuscript:

AI	Artificial Intelligence
HiL AI	Human-in-the-loop Artificial Intelligence
HoL AI	Human-out-of-the-loop Artificial Intelligence

## Appendix A. Semi Structured Interview (SSI) Protocol

### Introduce Self and Role

A. Confirm name and role of participant. Thank participant.
B. Ask participant for permission to record. If “yes”, record.
C. Review informed consent material
D. Confirm participant’s understanding of study protocol
E. Ask if any questions or clarification. Indicate interview will begin
  1. Can you tell me a little bit about your background, in pharmacy and in regulatory work?
  2. What has your personal experience been with use of AI in pharmacy practice?
  3. How would you describe the state of evolution of AI within pharmacy?
  4. Within your regulatory body, what have been some of the conversations and concerns that have been discussed with regards to AI in pharmacy practice? What kinds of conversations have you been hearing about amongst other pharmacy regulators?
  5. Within your regulatory body, what work has been undertaken with respect to regulation of AI in pharmacy practice? How would you describe the processes that are used by the regulatory body to identify priorities, build consensus, and seek validation?
  6. Based on your experience, how do you believe regulators view their role with respect to regulation of AI in pharmacy practice? [Prompt to expand/explain further]
  7. What have been some of the priorities from a regulatory perspective that your regulatory body has identified with respect to action? [Prompt to differentiate regulatory vs. educational vs. other approaches]
  8. What have been some of the areas that your regulatory body has decided are not subject to regulation or regulatory body interests?
  9. How do you see the regulation of AI in pharmacy practice evolving in the next 12 months? The next 3 years?
  10. In the context of AI in pharmacy practice, how has your regulatory body identified and categorized potential and actual risks? To patients? To professionals? To the profession of pharmacy? How has this risk stratification shaped identification of priorities and helped to build an approach to the issue?
  11. How has your regulatory body managed issues related to workforce deskilling brought about by AI [expand on “deskilling” as needed]?
  12. Are there any other questions or points you would like to discuss that we haven’t already touched on today?
F. Thank participant for involvement in interview
G. Confirm with participant their opportunity to review transcripts of interview if desired.
H. Indicate recording will be stopped. Stop recording
I. Ask participant if any further question, concerns, observations
J. Thank participant for involvement and conclude interview.
